# Loneliness, Chronic Conditions, and Healthcare Engagement in Japan: A Nationwide Cross-Sectional Study

**DOI:** 10.31662/jmaj.2026-0048

**Published:** 2026-05-22

**Authors:** Hiroshi Ito, Shuichi Okaya, Tatsuya Watanabe, Ying-Fang Zheng, Nobutake Shimojo, Satoru Kawano, Takahiro Tabuchi

**Affiliations:** 1 Department of Hospital Medicine, University of Tsukuba Hospital, Tsukuba, Japan; 2 Health Services Research and Development Center, University of Tsukuba, Tsukuba, Japan; 3 Department of Emergency and Critical Care Medicine, University of Tsukuba Hospital, Tsukuba, Japan; 4 Department of Internal Medicine, Ibaraki Prefectural University of Health Sciences Hospital, Inashiki, Japan; 5 Division of Epidemiology, School of Public Health, Tohoku University Graduate School of Medicine, Sendai, Japan

**Keywords:** loneliness, health outcomes, UCLA 3-Item Loneliness Scale, healthcare-seeking behaviors, chronic diseases

## Abstract

**Introduction:**

Loneliness has been increasingly recognized as a determinant of adverse health outcomes, including mortality and cardiovascular disease, primarily based on evidence from Western countries. However, data from East Asian populations using standardized loneliness measures remain limited. This study aimed to examine the associations between loneliness, assessed using the University of California, Los Angeles (UCLA) 3-Item Loneliness Scale, and multiple health-related outcomes among adults in Japan.

**Methods:**

We conducted a cross-sectional analysis using data from a nationwide, internet-based panel survey of the general population in Japan conducted in 2024. Participants aged 18-79 years were included. Loneliness was defined using the UCLA 3-Item Loneliness Scale. Primary outcomes were poor self-rated health and high symptom burden, assessed using the Somatic Symptom Scale-8. Weighted multivariable logistic regression models were used to estimate associations, adjusting for demographic, socioeconomic, and health-related factors, with additional adjustment for psychological distress.

**Results:**

Among 25,014 participants (mean [standard deviation {SD}] age, 50.0 [17.4] years; 49.9% men), 12,582 (50.3%) were classified as lonely. Loneliness was significantly associated with poor self-rated health (adjusted odds ratio [AOR], 2.71; 95% confidence interval [CI], 2.40-3.07) and high symptom burden (AOR, 3.61; 95% CI, 3.21-4.05). Loneliness was also associated with a higher prevalence of self-reported chronic diseases after adjusting for demographic, socioeconomic, and health-related factors; these associations were attenuated after further adjustment for psychological distress. In addition, lonely participants were less likely to report having a family doctor and demonstrated different patterns of engagement with preventive healthcare services.

**Conclusions:**

Loneliness was associated with poorer perceived health, greater symptom burden, and altered healthcare-seeking behaviors, with attenuation after adjustment for psychological distress. These findings suggest that loneliness reflects health-relevant vulnerability beyond traditional risk factors and underscore the importance of addressing loneliness within population health strategies, even in healthcare systems with high accessibility.

## Introduction

Loneliness has increasingly been recognized as an important social determinant of health and well-being ^(1)^, affecting a substantial proportion of the population in many countries ^(2), (3), (4)^. Previous studies, conducted mainly in Western countries, have reported associations between loneliness and increased risk of all-cause mortality, including deaths from cancer and cardiovascular disease, as well as a range of adverse mental health outcomes such as depression and anxiety ^(5), (6)^. These associations may be mediated through multiple, interrelated pathways, including biological processes (such as neuroendocrine and immune dysregulation), behavioral changes (such as reduced engagement in health-promoting behaviors), and psychological stress responses ^(7), (8)^. In light of these broad health implications, the World Health Organization has identified loneliness and social disconnection as emerging global public health concerns and has called for comprehensive actions to strengthen social connections ^(9)^.

Despite the growing interest in the health effects of loneliness, several limitations remain in the existing epidemiological literature. Much of the prior research has focused on objective endpoints such as mortality and cardiovascular events ^(5), (10), (11), (12), (13)^, whereas associations with more proximal indicators of health burden in daily life―including patient-reported health and symptom burden, as well as the broader prevalence of chronic conditions―have been less thoroughly examined ^(14), (15)^. In addition, individuals experiencing loneliness may be less likely to engage in healthcare-seeking behaviors, such as visiting medical institutions or utilizing preventive health services, even when they are aware of health problems ^(16)^. These issues may be underestimated in studies that rely solely on diagnosis-based outcomes, without considering patient perspectives and healthcare-seeking behaviors. Furthermore, much of the existing evidence on the association between loneliness and health has been derived from Western countries, and its generalizability to East Asian populations, which differ in social structure and cultural context ^(17), (18), (19)^, remains uncertain. In Asian countries, prior studies have often relied on measures of depressive symptoms as proxy indicators of loneliness, whereas large-scale epidemiological studies using standardized instruments that directly assess loneliness are limited ^(20), (21), (22)^. Therefore, there is a clear knowledge gap regarding the comprehensive evaluation of the associations between loneliness and a broad range of health-related outcomes in non-Western settings.

Japan provides a unique context in which to examine these associations. Under a universal health coverage system with a free-access model, healthcare barriers at the system level are relatively low ^(23), (24)^. However, even in this setting of high healthcare accessibility, it remains unclear how loneliness relates to multiple dimensions of health and healthcare engagement, beyond specific outcomes such as coronavirus disease 2019 (COVID-19) infection and hospitalization ^(25), (26)^. Therefore, in this study, we used the University of California, Los Angeles (UCLA) 3-Item Loneliness Scale to assess loneliness in a large, nationwide, internet-based survey of the general adult population in Japan. We examined the associations between loneliness and a range of health-related outcomes, including subjective health status, symptom burden, chronic disease prevalence, and healthcare-seeking behaviors.

## Materials and Methods

### Study design and setting

This cross-sectional analysis used data from the Japan COVID-19 and Society Internet Survey (JACSIS), a nationwide, online, self-administered survey implemented by Rakuten Insight, Inc., one of Japan’s leading internet research firms ^(27)^. During the study period, Rakuten Insight operated a large survey panel comprising approximately 2.2 million registered members nationwide. Study participants were selected in cooperation with the company using sampling procedures intended to reflect the demographic characteristics of the general Japanese population. Data from the JACSIS have been extensively utilized in prior epidemiological research ^(25), (26)^.

The 2024 wave of the JACSIS gathered data on sociodemographic attributes, lifestyle behaviors, health conditions, and healthcare use from approximately 28,000 respondents aged 15-79 years. For this analysis, we excluded participants aged 15-17 years, those who did not pass an embedded attention-check item, respondents who indicated use of all queried illicit substances, and those who reported having all listed medical conditions, as such response patterns were deemed unreliable (Supplementary Table 1). Since the analytic dataset included individuals newly enrolled in the survey panel, an overall response rate for the target population could not be calculated.

The study protocol was reviewed and approved by the Research Ethics Committee of the Osaka International Cancer Institute (approval no. 20084-2) before data collection, and was later amended and approved by the Ethics Committee of the University of Tsukuba Hospital (approval no. R07-178). All study procedures complied with the principles of the Declaration of Helsinki, and the reporting of this study follows the Strengthening the Reporting of Observational Studies in Epidemiology (STROBE) guidelines.

### Exposure variable: loneliness

The primary exposure was loneliness, assessed using the UCLA 3-Item Loneliness Scale ^(28)^. The UCLA 3-Item Loneliness Scale is a validated brief instrument designed to measure subjective feelings of loneliness, including perceived lack of companionship, feeling left out, and feeling isolated from others. The scale has been translated and validated in Japanese populations and is widely used in epidemiological studies ^(29), (30)^.

In the 2024 survey, respondents were asked how often during the past 30 days they experienced each of the three loneliness-related feelings. Response options for each item were “never,” “rarely,” “sometimes,” and “often.” Each item was scored on a three-point scale (1 = never or rarely, 2 = sometimes, and 3 = often), with higher scores indicating greater loneliness. The total loneliness score was calculated by summing the three items, resulting in a possible range from 3 to 9 (Supplementary Table 2). Consistent with prior studies ^(29), (30)^, participants with a score of 6 or higher were classified as lonely, whereas those with a score below 6 were classified as not lonely. This cutoff has been widely used in population-based studies to identify individuals experiencing frequent feelings of loneliness. For instance, a nationwide survey conducted in Japan during the COVID-19 pandemic reported that approximately 41% of respondents were classified as lonely using the same cutoff ^(31)^. In addition, we explored potential dose-response relationships using alternative categorizations of the loneliness score in sensitivity analyses.

### Outcome variables

The primary outcomes were self-rated health and symptom burden assessed at the time of the survey. Self-rated health was measured using a commonly employed single-item question. In the 2024 survey, participants were asked, “How would you describe your current health status? Please select the one option that best applies to you.” Poor self-rated health was defined as responses of “Not very good” or “Not good,” whereas respondents who selected “Good,” “Fairly good,” or “Average” were categorized as not having poor self-rated health.

Symptom burden was assessed using the Somatic Symptom Scale-8 (SSS-8), a validated instrument for evaluating the severity of somatic symptoms, which has also been validated in Japanese populations ^(32), (33)^. Participants were asked, “During the past week, how much have you been bothered by the following physical symptoms?” The eight symptoms included: stomach or bowel problems; back pain; pain in the arms, legs, or joints; headaches; chest pain or shortness of breath; dizziness; fatigue or low energy; and sleep difficulties. Each symptom was rated on a five-point Likert scale from 0 (“Not at all”) to 4 (“Very much”), resulting in a total score ranging from 0 to 32. Consistent with established thresholds, a total SSS-8 score of 13 or higher was used to define high symptom burden.

Secondary outcomes comprised physician-diagnosed chronic conditions and healthcare-seeking behaviors. Chronic conditions were identified based on self-reported physician diagnoses and included hypertension, diabetes mellitus, dyslipidemia, coronary artery disease, stroke, cancer, chronic kidney disease, chronic obstructive pulmonary disease, asthma, atopic dermatitis, liver disease, and mental disorders. Participants were considered to have a given condition if they indicated that it was “Present now,” while those selecting “Not present” or “Not present now but in the past” were classified as not having the condition. Healthcare-seeking behaviors included having a family doctor, undergoing routine health checkups, receiving dental checkups, and having been vaccinated in the previous year. All healthcare-seeking behaviors were assessed as binary variables (yes/no). In Japan, there is no formal gatekeeping system or registered general practitioner system. Therefore, “having a family doctor” in this survey referred to whether respondents reported having a physician whom they usually consult for health concerns.

### Covariates

We accounted for a set of demographic, socioeconomic, and lifestyle-related factors that could act as potential confounders of the association between loneliness and health outcomes. For each outcome, weighted multivariable logistic regression analyses were performed using a stepwise modeling approach. Model 1 included demographic and socioeconomic variables. Model 2 further incorporated lifestyle-related factors, and Model 3 additionally adjusted for psychological distress. Since psychological distress may be closely intertwined with loneliness and may function as either a confounder or a mediator, Model 3 should be interpreted as an exploratory model intended to examine how adjustment for psychological distress influences the observed associations.

Demographic characteristics comprised age (categorized in 10-year intervals), sex, and region of residence. Residential region was classified into eastern Japan (Hokkaido, Tohoku, and Kanto), central Japan (Chubu and Kinki), and western Japan (Chugoku, Shikoku, Kyushu, and Okinawa). Socioeconomic variables included educational attainment (college or higher vs high school or lower), equivalent household income, occupational class (upper non-manual workers, lower non-manual workers, manual workers, farmers, self-employed individuals, and unemployed individuals) ^(34)^, marital status (married, unmarried, widowed, or separated), and household size (1, 2, 3, 4, or ≥5 members). Equivalent household income was calculated by dividing total household income by the square root of household size and categorized into quintiles, with an additional category for unanswered responses. These variables were selected based on commonly used socioeconomic and health-related indicators in epidemiological research, with consideration given to their availability in the JACSIS and their potential role as confounders in the relationship between loneliness and health outcomes ^(35), (36), (37)^.

Lifestyle-related variables included body mass index (<18.5, 18.5-24.9, and ≥25.0 kg/m^2^), smoking status (never, ever, or current), alcohol consumption (never, <20 g/day, 20-40 g/day, or ≥40 g/day), daily walking time (<60 minutes/day), exercise duration (<30 minutes/day), and sleep duration (<6 hours/day). Cutoff values for these variables were defined in accordance with recommendations from Japan’s national health promotion initiative, Health Japan 21 ^(38)^.

Psychological distress was measured using the Kessler Psychological Distress Scale (K6) ^(39)^, which has been validated in Japanese populations ^(40)^. Participants reported how frequently they had experienced the following six symptoms during the past 30 days: feeling nervous; feeling hopeless; feeling restless or fidgety; feeling so depressed that nothing could cheer them up; feeling that everything was an effort; and feeling worthless. Response options ranged from “none of the time” to “all of the time” and were scored from 0 to 4, respectively. Total K6 scores ranged from 0 to 24, with higher scores indicating greater levels of psychological distress. For covariate adjustment, K6 scores were dichotomized into higher (≥13) and lower (<13) distress categories to reduce multicollinearity and to enhance interpretability of the regression models.

### Statistical analysis

First, we summarized characteristics of participants according to loneliness. To address potential selection bias inherent in internet-based surveys, we applied inverse probability weighting (IPW), using demographic distributions from the 2019 Comprehensive Survey of Living Conditions as the reference population ^(41)^. The weighting procedure was designed to align the distribution of key demographic characteristics in the analytic sample with those of the general Japanese population (detailed descriptions of the methods presented elsewhere) ^(42)^. This approach has been widely used in previous studies based on the JACSIS dataset ^(25), (26)^. We then examined associations between loneliness and each outcome using weighted multivariable logistic regression models. All models were adjusted for the covariates described above. Adjusted odds ratios (AORs) and 95% confidence intervals (CIs) were calculated, with socially connected participants serving as the reference group. A two-sided p value of <0.05 was considered statistically significant. All analyses were conducted using Stata version 15 (StataCorp LLC, College Station, TX, USA). Missing data were minimal because most survey items in the JACSIS questionnaire require responses before submission.

### Sensitivity analyses

As part of the sensitivity analyses, all regression models were re-estimated without IPW (i.e., using unweighted models) to evaluate the stability of the results with respect to the weighting approach. In further analyses, potential dose-response associations between loneliness and the primary outcomes were explored by applying alternative categorizations of the UCLA 3-Item Loneliness Scale score. Specifically, inverse probability-weighted logistic regression models were fitted to estimate the associations between each score category (3 [reference], 4, 5, 6, 7, 8, and 9) and the primary outcomes, with adjustment for Model 1 covariates. The resulting estimates were displayed graphically using forest plots.

### Patient and public involvement

Participants were not involved in the design, conduct, reporting, or dissemination plans of this research. Informed consent was obtained electronically from all participants prior to their participation.

## Results

### Participant characteristics

Among the 28,000 respondents to the 2024 JACSIS, 25,014 individuals aged 18-79 years were retained for the analytic sample after excluding those aged 15-17 years and respondents with invalid or implausible answers (Supplementary Figure 1). The participants had a mean (standard deviation [SD]) age of 50.0 (17.4) years, and 49.9% were male (Table 1). Overall, 12,582 participants (50.3%) were classified as lonely based on the UCLA 3-Item Loneliness Scale. Compared with participants without loneliness, those classified as lonely were more likely to be unmarried, to live alone, to have lower equivalent household income, and to be unemployed. They also exhibited a higher prevalence of adverse lifestyle factors, including current smoking, insufficient physical activity, and short sleep duration. In addition, several self-reported chronic conditions were more prevalent among participants with loneliness.

**Table 1.** Characteristics of the Participants.

Characteristics	Participants with loneliness (n = 12,582)	Participants without loneliness (n = 12,432)
Male, n (%)	5,983 (47.6)	6,500 (52.3)
Age (years), mean (SD)	49.6 (17.4)	50.5 (17.5)
Age in 10-year intervals, n (%)		
18-29	2,020 (16.1)	1,882 (15.1)
30-39	2,222 (17.7)	2,019 (16.2)
40-49	2,114 (16.8)	2,129 (17.1)
50-59	2,224 (17.7)	2,106 (16.9)
60-69	1,937 (15.4)	2,047 (16.5)
70-79	2,065 (16.4)	2,249 (18.1)
Region, n (%)		
East Japan	4,811 (38.2)	4,941 (39.7)
Central Japan	4,538 (36.1)	4,505 (36.2)
West Japan	3,233 (25.7)	2,985 (24.0)
Educational attainment, n (%)		
College or higher	5,564 (44.2)	5,529 (44.5)
High school or lower	7,018 (55.8)	6,903 (55.5)
Equivalent household income, n (%)		
1st quintile (<1.8 million JPY)	1,842 (14.6)	1,324 (10.6)
2nd quintile (1.8-3.0 million JPY)	2,738 (21.8)	2,374 (19.1)
3rd quintile (3.0-4.0 million JPY)	1,693 (13.5)	1,831 (14.7)
4th quintile (4.0-5.5 million JPY)	1,761 (14.0)	2,005 (16.1)
5th quintile (≥5.5 million JPY)	1,310 (10.4)	1,722 (13.9)
Unanswered	3,238 (25.7)	3,176 (25.5)
Occupation, n (%)		
Upper non-manual workers	1,151 (9.1)	1,377 (11.1)
Lower non-manual workers	4,224 (33.6)	4,406 (35.4)
Manual workers	2,029 (16.1)	1,792 (14.4)
Farmers	127 (1.0)	83 (0.7)
Self-employed	704 (5.6)	777 (6.3)
Unemployed	4,346 (34.5)	3,997 (32.2)
Marital status, n (%)		
Married	7,529 (59.8)	8,398 (67.6)
Never married	3,542 (28.2)	2,867 (23.1)
Widowed	799 (6.4)	582 (4.7)
Separated	711 (5.7)	586 (4.7)
Household size, n (%)		
1	2,388 (19.0)	1,907 (15.3)
2	4,191 (33.3)	4,284 (34.5)
3	2,803 (22.3)	3,047 (24.5)
4	2,264 (18.0)	2,167 (17.4)
≥5	936 (7.4)	1,027 (8.3)
Body mass index (kg/m^2^), mean (SD)	22.6 (7.6)	22.6 (6.6)
Smoking, n (%)		
Never	2,034 (16.2)	4,017 (32.3)
Ever	6,138 (48.8)	5,260 (42.3)
Current	4,410 (35.1)	3,155 (25.4)
Alcohol, n (%)		
Never	6,583 (52.3)	6,164 (49.6)
≤20 g/day	2,796 (22.2)	2,938 (23.6)
20-40 g/day	1,761 (14.0)	1,732 (13.9)
>40 g/day	1,441 (11.5)	1,598 (12.9)
Walking <60 min/day, n (%)	5,897 (46.9)	5,431 (43.7)
Exercising <30 min/day, n (%)	9,641 (76.6)	9,446 (76.0)
Sleeping <6 hours/day, n (%)	3,618 (28.8)	2,721 (21.9)
Comorbidities, n (%)		
Hypertension	3,286 (26.1)	2,785 (22.4)
Periodontal diseases	2,525 (20.1)	1,597 (12.8)
Dyslipidemia	2,199 (17.5)	1,774 (14.3)
Dental caries	2,175 (17.3)	1,194 (9.6)
Diabetes mellitus	1,233 (9.8)	959 (7.7)
Mental diseases	1,430 (11.4)	378 (3.0)
Asthma	743 (5.9)	409 (3.3)
Coronary artery diseases	560 (4.5)	302 (2.4)
Malignancies	514 (4.1)	336 (2.7)
Stroke	382 (3.0)	174 (1.4)
COPD	374 (3.0)	120 (1.0)

We applied inverse probability weighting to account for selection into the internet survey and the exclusion of ineligible cases. COPD: chronic obstructive pulmonary diseases; JPY: Japanese yen; SD: standard deviation.

### Loneliness and subjective health outcomes

**Figure 1.**
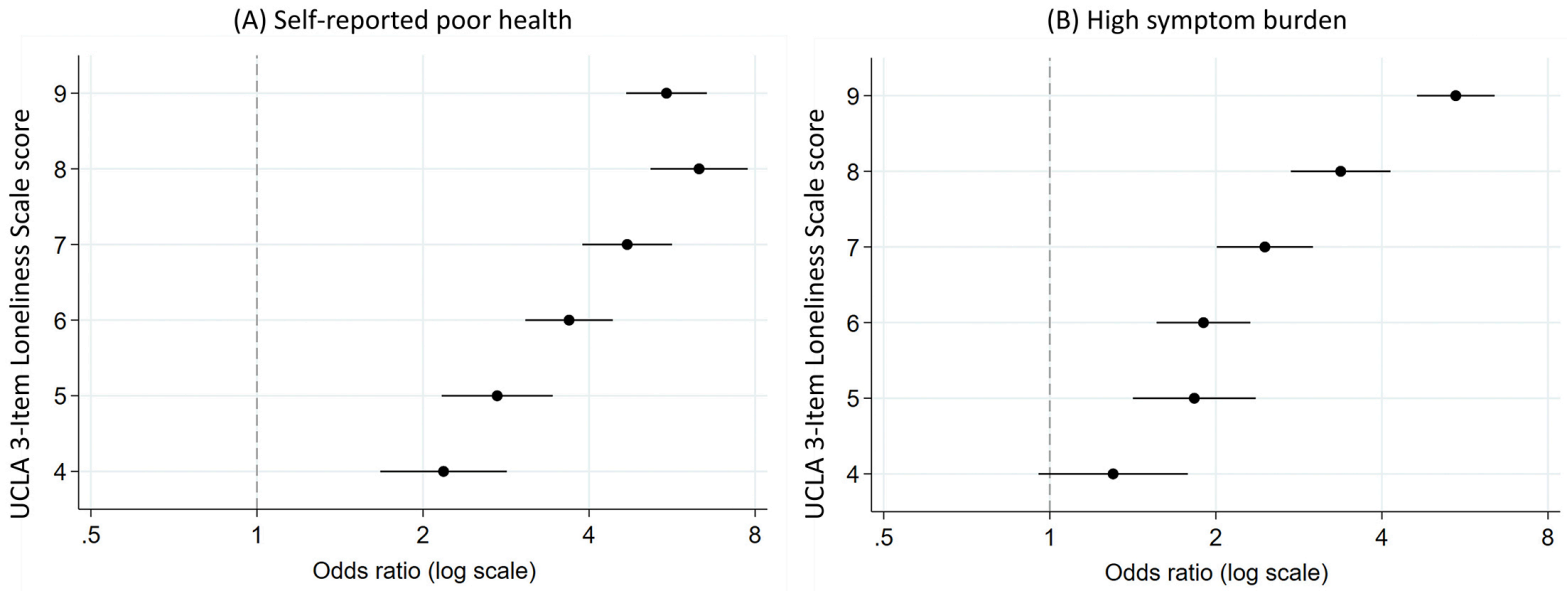
Association between the UCLA 3-Item Loneliness Scale score and subjective health status. The analyses applied inverse probability weighting to account for selection into the internet-based survey and the exclusion of ineligible cases. For each outcome, we constructed weighted multivariable logistic regression models adjusted for demographic and socioeconomic status. In this figure, the UCLA 3-Item Scale score was categorized into seven groups (3 [reference], 4, 5, 6, 7, 8, and 9) for visualization, in contrast to the primary analyses using a dichotomized measure (score ≥6 [lonely] vs <6 [not lonely]). A significant dose-response relationship was observed for both self-reported poor health and high symptom burden (p for trend <0.001 for both). UCLA: University of California, Los Angeles.

In weighted logistic regression analyses adjusted for demographic and socioeconomic factors (Model 1), loneliness was strongly associated with poor self-rated health (AOR, 2.71; 95% CI, 2.40-3.07) and high symptom burden (AOR, 3.61; 95% CI, 3.21-4.05) (Table 2). These associations remained statistically significant after additional adjustment for health-related status (Model 2). However, after further adjustment for psychological distress (Model 3), the associations were substantially attenuated and no longer statistically significant (AOR for poor self-rated health, 1.19; 95% CI, 0.98-1.45 and AOR for high symptom burden, 1.17; 95% CI, 0.99-1.39). When the UCLA 3-Item Loneliness Scale score was categorized into seven levels (3-9), a clear dose-response relationship was observed between increasing loneliness scores and both poor self-rated health and high symptom burden (p for trend <0.001 for both outcomes) (Figure 1).

**Table 2.** Association between Loneliness and Subjective Health Status.

	Participants with loneliness, n (%)	Participants without loneliness, n (%)	Model 1			Model 2			Model 3		
			AOR	95% CI	p Value	AOR	95% CI	p Value	AOR	95% CI	p Value
Self-reported poor health	2,317 (18.4)	956 (7.7)	2.71	2.40-3.07	<0.001	2.54	2.18-2.96	<0.001	1.19	0.98-1.45	0.08
High symptom burden*	3,585 (28.5)	1255 (10.1)	3.61	3.21-4.05	<0.001	3.06	2.71-3.47	<0.001	1.17	0.99-1.39	0.07

The analyses applied inverse probability weighting to account for selection into the internet-based survey and the exclusion of ineligible cases. For each outcome, we constructed weighted multivariable logistic regression models with stepwise adjustment. Model 1 adjusted for demographic and socioeconomic factors. Model 2 additionally adjusted for health-related status, and Model 3 further included psychological distress. AOR: adjusted odds ratio; CI: confidence interval. *High symptom burden was defined as Somatic Symptom Scale 8 score ≥13 points.

### Loneliness and chronic diseases

Participants with loneliness were more likely to have several self-reported chronic conditions in models adjusted for demographic and socioeconomic factors (Table 3). In fully adjusted models including psychological distress (Model 3), loneliness remained significantly associated with periodontal disease (AOR, 1.49; 95% CI, 1.29-1.72), dental caries (AOR, 1.58; 95% CI, 1.35-1.86), and mental disorders (AOR, 1.28; 95% CI, 1.01-1.62). In contrast, associations with cardiometabolic conditions, such as hypertension, diabetes mellitus, dyslipidemia, coronary artery disease, and stroke, were attenuated and no longer statistically significant after full adjustment.

**Table 3.** Association between Loneliness and Self-Reported Comorbidities.

	Participants with loneliness, n (%)	Participants without loneliness, n (%)	Model 1			Model 2			Model 3		
			AOR	95% CI	p Value	AOR	95% CI	p Value	AOR	95% CI	p Value
Hypertension	3,286 (26.1)	2,785 (22.4)	1.43	1.29-1.59	<0.001	1.33	1.19-1.49	<0.001	1.12	0.98-1.28	0.10
Periodontal diseases	2,525 (20.1)	1,597 (12.8)	1.77	1.58-1.99	<0.001	1.62	1.44-1.82	<0.001	1.49	1.29-1.72	<0.001
Dyslipidemia	2,199 (17.5)	1,774 (14.3)	1.39	1.24-1.56	<0.001	1.28	1.14-1.45	<0.001	1.09	0.94-1.26	0.26
Dental caries	2,175 (17.3)	1,194 (9.6)	2.00	1.76-2.26	<0.001	1.77	1.56-2.02	<0.001	1.58	1.35-1.86	<0.001
Diabetes mellitus	1,233 (9.8)	959 (7.7)	1.44	1.24-1.68	<0.001	1.24	1.06-1.46	0.008	1.08	0.89-1.32	0.44
Mental diseases	1,430 (11.4)	378 (3.0)	3.95	3.27-4.77	<0.001	3.22	2.66-3.90	<0.001	1.28	1.01-1.62	0.04
Asthma	743 (5.9)	409 (3.3)	1.83	1.51-2.22	<0.001	1.55	1.28-1.88	<0.001	1.08	0.83-1.39	0.57
Coronary artery diseases	560 (4.5)	302 (2.4)	2.01	1.54-2.61	<0.001	1.65	1.25-2.18	<0.001	1.12	0.77-1.63	0.55
Malignancies	514 (4.1)	336 (2.7)	1.65	1.32-2.07	<0.001	1.34	1.05-1.70	0.02	0.93	0.71-1.22	0.61
Stroke	382 (3.0)	174 (1.4)	2.27	1.67-3.09	<0.001	1.75	1.29-2.39	<0.001	0.92	0.58-1.45	0.73
COPD	374 (3.0)	120 (1.0)	3.33	2.36-4.70	<0.001	2.40	1.67-3.45	<0.001	1.56	0.91-2.66	0.10

The analyses applied inverse probability weighting to account for selection into the internet-based survey and the exclusion of ineligible cases. For each outcome, we constructed weighted multivariable logistic regression models with stepwise adjustment. Model 1 adjusted for demographic and socioeconomic factors. Model 2 additionally adjusted for health-related status, and Model 3 further included psychological distress. AOR: adjusted odds ratio; CI: confidence interval; COPD: chronic obstructive pulmonary diseases.

### Loneliness and healthcare-seeking behaviors

Across all models with adjustment, the associations between loneliness and healthcare-seeking behaviors were generally consistent (Table 4). In fully adjusted models, participants with loneliness were less likely to have a family doctor (AOR, 0.85; 95% CI, 0.77-0.95). In contrast, they were more likely to receive annual health checkups (AOR, 1.28; 95% CI, 1.13-1.45) and vaccination in the previous year (AOR, 1.11; 95% CI, 1.00-1.24).

**Table 4.** Association between Loneliness and Healthcare-Seeking Behaviors.

	Participants with loneliness, n (%)	Participants without loneliness, n (%)	Model 1			Model 2			Model 3		
			AOR	95% CI	p Value	AOR	95% CI	p Value	AOR	95% CI	p Value
Having a family doctor	5,803 (46.1)	6,374 (51.3)	0.84	0.77-0.91	<0.001	0.85	0.78-0.93	<0.001	0.85	0.77-0.95	0.004
Receiving annual health checkups	8,911 (70.8)	8,914 (71.7)	1.06	0.97-1.17	0.20	1.16	1.05-1.28	0.004	1.28	1.13-1.45	<0.001
Receiving annual dental checkups	7,111 (56.5)	7,317 (58.9)	0.95	0.87-1.03	0.21	0.99	0.91-1.08	0.91	0.95	0.85-1.05	0.30
Vaccination in the previous year	5,712 (45.4)	5,833 (46.9)	1.01	0.93-1.10	0.79	1.05	0.96-1.15	0.27	1.11	1.00-1.24	0.047

The analyses applied inverse probability weighting to account for selection into the internet survey and the exclusion of ineligible cases. For each outcome, we constructed weighted multivariable logistic regression models with stepwise adjustment. Model 1 adjusted for demographic and socioeconomic factors. Model 2 additionally adjusted for health-related status, and Model 3 further included psychological distress. AOR: adjusted odds ratio; CI: confidence interval.

### Sensitivity analyses

In sensitivity analyses using unweighted regression models, the overall patterns of association between loneliness and subjective health outcomes, chronic diseases, and healthcare-seeking behaviors were similar to those observed in the primary weighted analyses (Supplementary Table 3-5).

## Discussion

In this nationwide cross-sectional study of the general adult population in Japan, we comprehensively examined the associations between loneliness and a range of health-related outcomes. Our findings highlight two distinctive patterns. First, the associations between loneliness and subjective health outcomes were substantially attenuated after adjustment for psychological distress, suggesting that psychological distress may play an important role in explaining the relationship between loneliness and perceived health. Second, individuals experiencing loneliness exhibited distinctive patterns of healthcare engagement, including a lower likelihood of having a regular source of care despite relatively frequent use of certain preventive services. Loneliness was associated with poorer self-rated health and greater symptom burden in models adjusted for demographic, socioeconomic, and health-related factors, whereas associations with the prevalence of chronic diseases were less consistent. Notably, these associations were observed in Japan, a country characterized by relatively high healthcare accessibility under a universal health coverage system, underscoring the importance of considering loneliness in understanding population health.

Our finding that loneliness was associated with poorer self-rated health and greater symptom burden is broadly consistent with prior epidemiological evidence, derived mainly from Western countries, showing that loneliness is linked to lower levels of activities of daily living, sensory impairments, anxiety and depression, and other mental health-related symptoms such as insomnia ^(43), (44)^. These associations may reflect multiple, interrelated mechanisms operating through psychosocial and behavioral pathways; loneliness can function as a chronic psychosocial stressor, activating the hypothalamic-pituitary-adrenal axis and contributing to dysregulation of autonomic and immune function, thereby amplifying nonspecific somatic symptoms ^(45), (46), (47)^. In addition, individuals experiencing loneliness may be more prone to adverse health-related behaviors, including reduced physical activity and poorer sleep quality, which may further contribute to poorer perceived health ^(48), (49)^. In our study, the associations between loneliness and both subjective health and symptom burden were attenuated after additionally adjusting for psychological distress. This finding suggests that psychological distress may partially mediate the relationship between loneliness and these outcomes, or alternatively, may capture shared underlying stress-related processes that contribute to both loneliness and adverse health. Because psychological distress is closely intertwined with loneliness, adjustment for K6 may also represent partial overadjustment if psychological distress lies on the causal pathway between loneliness and health outcomes. Therefore, the attenuation observed in Model 3 should be interpreted cautiously, and may reflect shared underlying psychological processes rather than purely confounding effects. At the same time, the relationship among loneliness, psychological distress, and health outcomes may be bidirectional. Poor health or chronic symptoms may increase psychological distress and social withdrawal, which in turn may intensify feelings of loneliness. Conversely, loneliness may contribute to psychological distress and negative health perceptions through chronic stress pathways. Due to the cross-sectional design, this study cannot disentangle these potential bidirectional processes.

In contrast to its strong associations with subjective health outcomes, loneliness showed comparatively weak associations with chronic conditions, which tended to be attenuated or disappear after adjustment for psychological distress. Although loneliness may alter patterns of healthcare utilization, such changes do not necessarily translate into increased opportunities for the diagnosis of chronic diseases ^(50), (51)^. Individuals experiencing loneliness may exhibit non-specific healthcare use driven by psychological stress or anxiety, while simultaneously being less likely to maintain continuous relationships with healthcare providers, such as having a regular source of care. Asymptomatic chronic conditions, such as hypertension or diabetes, may be less likely to be detected and formally diagnosed in such situations ^(52), (53)^. The persistence of associations between loneliness and dental as well as mental disorders, after adjustment for psychological distress, suggests that these outcomes may be less dependent on diagnostic opportunity and more directly linked to social disconnection and reduced self-care behaviors, such as poorer oral hygiene, delayed dental visits, or reduced motivation to seek mental health support, which are core features of loneliness ^(54)^.

Taken together, these findings suggest that even in a high-access healthcare environment such as Japan, characterized by universal health coverage and a free-access system, loneliness is associated with differences in how individuals engage with healthcare; individuals experiencing loneliness tended to use health checkups and some preventive services but were less likely to have a regular source of care. In Japan, annual health checkups are commonly provided through employers or municipal programs and may therefore occur independently of individuals’ active healthcare-seeking behavior. This suggests that their healthcare utilization may be fragmented, lacking continuity or integration over time. In addition to the institutional feature of unrestricted access to medical care, this pattern may reflect structural constraints in Japan, where primary care physicians account for only 1% of the physician workforce, which may further contribute to fragmented patterns of care ^(55)^. Notably, even in the United States, where the proportion of primary care physicians is higher than in Japan ^(56)^, recent reports have suggested shortages of physicians accepting new patients ^(57)^, indicating that access to continuous care is not guaranteed even in systems with a more established primary care structure. Our findings suggest that health-related vulnerability may exist beyond what can be captured by diagnosis-based disease indicators alone, and that incorporating the assessment of loneliness may be informative for both clinical practice and public health.

This study has several limitations. First, the use of an internet-based panel survey may limit generalizability. Participants were individuals with access to and willingness to respond to online surveys, characteristics that could be associated with both loneliness and health-related behaviors, potentially influencing the prevalence and nature of loneliness observed in this study ^(58)^. In addition, as participation in online panels is voluntary, selection bias may occur if individuals who are more socially engaged or digitally connected are more likely to participate in the study. Individuals with limited internet access or lower digital literacy may therefore be underrepresented. Although IPW was applied to reduce selection bias, residual differences from the general population may remain. Second, most exposures, outcomes, and covariates were self-reported, which introduces the possibility of reporting and recall biases as well as misclassification. In addition, somatic symptom reporting may be influenced by negative affectivity or psychological distress, which could lead to elevated SSS-8 scores, independent of actual physical symptom burden. At the same time, the inclusion of patient-reported health status and symptom burden represents an important strength of this study, capturing health impacts that may not be reflected in diagnosis-based indicators alone. Third, the cross-sectional design precludes causal inference and makes it difficult to disentangle temporality and bidirectionality; loneliness may contribute to poorer health and altered healthcare engagement, but existing health problems could also increase loneliness. Furthermore, this study cannot determine whether loneliness is prospectively associated with hard clinical endpoints such as cardiovascular events or mortality. Finally, unmeasured and residual confounding is possible despite comprehensive adjustment, including psychological distress, and future longitudinal studies with objective clinical measures are warranted to clarify causal pathways and mechanisms.

### Conclusions

Loneliness may be associated with the processes through which health problems are perceived, recognized, and diagnosed, as well as with health status. Our findings suggest that even in countries with high levels of healthcare access, such as Japan, there may be populations whose health-related vulnerabilities remain insufficiently identified through conventional, diagnosis-based approaches. Addressing loneliness may be a relevant consideration in more comprehensive strategies to improve population health.

## Article Information

### Acknowledgments

The data used in this study were originally collected under the project (JACSIS 2024) supported by the Japan Society for the Promotion of Science (JSPS) KAKENHI Grants (grant number JP21H04856; JP25H01079 [Dr. Takahiro Tabuchi]; and JP24K15576 [Dr. Kanami Tsuno]), JST RISTEX grants (grant number JPMJRX21K6) (Dr. Midori Matsushima), the intramural fund of the National Institute for Environmental Studies (Dr. Shoji Nakayama), the Health Labor Sciences Research Grant 23EA1003 (Dr. Masayoshi Zaitsu); 22FA2001 (Dr. Daisuke Nishi); 24LA0201 (Dr. Atsushi Nakagomi); 22JA1005; 22FA1001; 22FA1010; 23EA1001; 23FA1004; 24FA1020; 24LA1002 (Dr. Takahiro Tabuchi), and the Japan Agency for Medical Research and Development “Strategic Center of Biomedical Advanced Vaccine Research and Development for Preparedness and Response (grant number JP243fa627004)” (Dr. Yuki Furuse).

### Author Contributions

Hiroshi Ito: Conceptualization; Methodology; Data curation; Formal analysis; Investigation; Writing―original draft; Writing―review & editing; Funding acquisition; Project administration. Shuichi Okaya, Tatsuya Watanabe, Ying-Fang Zheng, Nobutake Shimojo, and Satoru Kawano: Methodology; Investigation; Writing―review & editing. Takahiro Tabuchi: Resources; Supervision; Writing―review & editing. All authors approved the final manuscript and agreed to be accountable for all aspects of the work.

### Conflicts of Interest

Dr. Takahiro Tabuchi received financial support for research (research fundings, consulting fees, or lecture fees) from Daiichi Sankyo Healthcare Co., Ltd., Johnson & Johnson K.K., Data Seed Inc., Workout-Plus LLC, and EMMA Co., Ltd. Other authors declare that there are no conflicts of interest.

### Ethical Approval Code

The study protocol was reviewed and approved by the Research Ethics Committee of the Osaka International Cancer Institute (no. 20084-2) prior to survey initiation, and was subsequently updated and approved by the Ethics Committee of the University of Tsukuba Hospital (R07-178).

## Supplement

Supplementary Material
