# Supplementary material for: Loneliness, Chronic Conditions, and Healthcare Engagement in Japan: A Nationwide Cross-Sectional Study
Source: JMA J. 2026 May 22;9(4):804–14. doi: 10.31662/jmaj.2026-0048 (PMC13453461; doi:10.31662/jmaj.2026-0048)
Supplement: Supplementary file 1 [file 2433-3298-9-4-0804-s001.pdf]

Supplement

eFigure 1: Selection of the participants

eTable 1: Definition of invalid responses in the 2024 JACSIS survey

eTable 2: The UCLA 3-Item Loneliness Scale

eTable 3: Association between loneliness and subjective health status (unweighted version)

eTable 4: Association between loneliness and self-reported comorbidities (unweighted version)

eTable 5: Association between social isolation and healthcare seeking behaviors (unweighted version)

eFigure 1. Selection of the participants

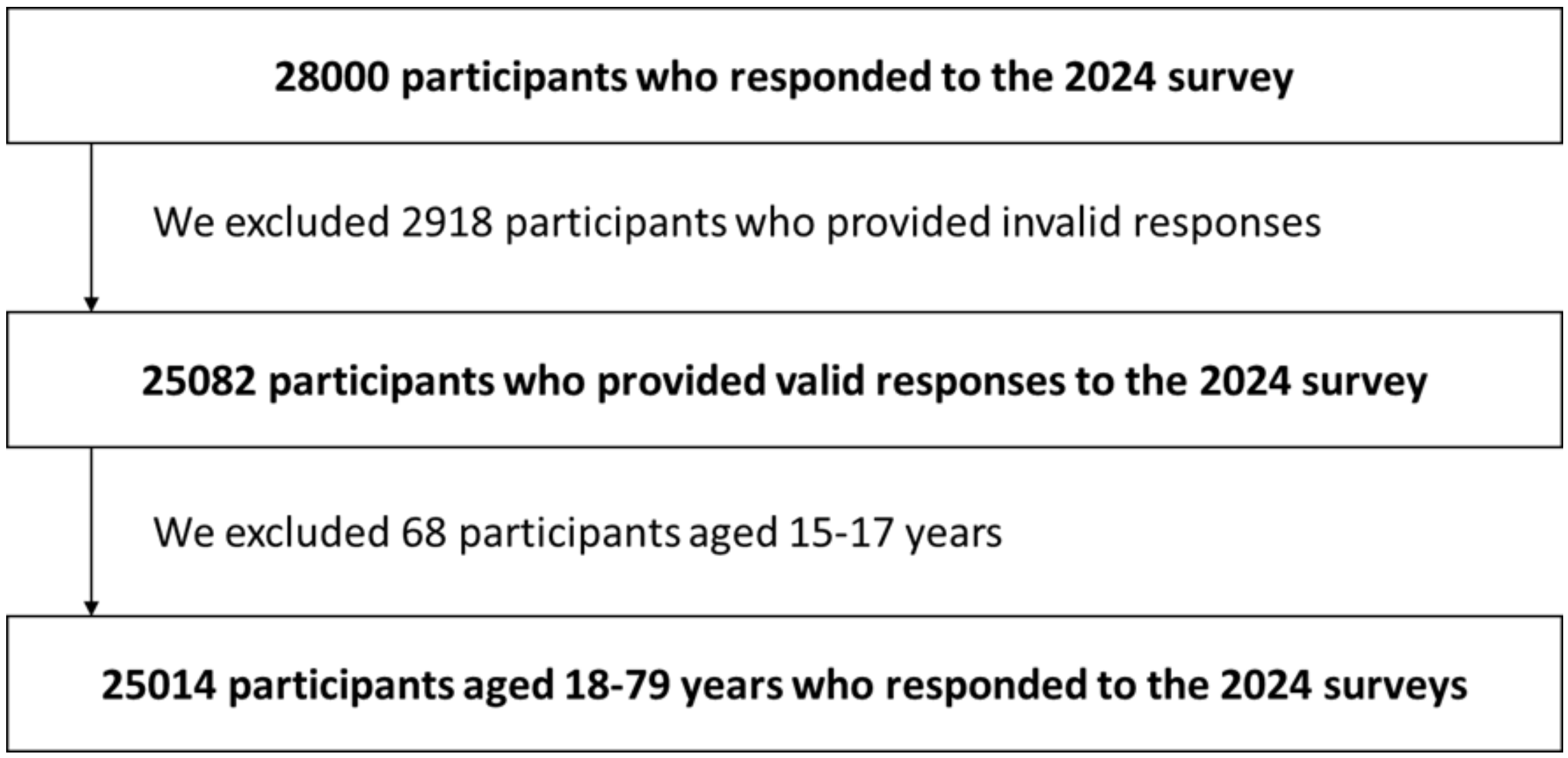

eTable 1. Definition of invalid responses in the 2024 JACSIS survey

**Inaccurate responses include following three definitions:**
1) those who selected "yes" to all disease lists (do you have the following diseases? (more than ten items listed below): hypertension, diabetes, asthma, bronchitis and pneumonia, atopic dermatitis, periodontal disease, caries, otitis media, coronary artery disease, stroke, chronic obstructive pulmonary disease), cancer, chronic pain such as back pain and headaches, depression, psychiatric disorders other than depression
and/or
2) those who selected "yes" to all drug use (do you use the following drugs?: alcohol, sleeping pills and/or anti-anxiety medications, morphine (three types), thinner, marijuana, dangerous drugs (e.g., outlawed herbs, magic mushrooms, etc.) and cocaine and/or heroin (nine items))
and/or
3) those who selected other than "D" out of "A B C D E" in the question of "please select the second option from the bottom."

eTable 2. The UCLA 3-Item Loneliness Scale

**How often do you feel that you lack companionship?**
Hardly ever or never (1 point)<br>Some of the time (2 points)<br>Often (3 points)
**How often do you feel left out?**
Hardly ever or never (1 point)<br>Some of the time (2 points)<br>Often (3 points)
**How often do you feel isolated from others?**
Hardly ever or never (1 point)<br>Some of the time (2 points)<br>Often (3 points)

The total score on the loneliness scale ranges from 3, representing a lower frequency of loneliness, to 9, representing a higher frequency.

eTable 3. Association between loneliness and subjective health status (unweighted version)

	Participants with loneliness, n (%)	Participants without loneliness, n (%)	Model 1			Model 2			Model 3		
			AOR	95% CI	p value	AOR	95% CI	p value	AOR	95% CI	p value
Self-reported poor health	3178 (25.2)	1120 (9.0)	3.14	2.92-3.39	<0.001	2.71	2.44-3.01	<0.001	1.26	1.11-1.44	<0.001
High symptom burden	3749 (29.7	1254 (10.1)	3.57	3.32-3.84	<0.001	2.95	2.73-3.18	<0.001	1.07	0.97-1.18	0.16

In this sensitivity analysis, we used unweighted regression instead of weighted regression; otherwise, see the caption of Table 1.

eTable 4. Association between loneliness and self-reported comorbidities (unweighted version)

	Participants with loneliness, n (%)	Participants without loneliness, n (%)	Model 1			Model 2			Model 3		
			AOR	95% CI	p value	AOR	95% CI	p value	AOR	95% CI	p value
Hypertension	3009 (23.8)	2684 (21.7)	1.48	1.38-1.58	<0.001	1.34	1.25-1.44	<0.001	1.10	1.02-1.20	0.02
Periodontal diseases	2386 (18.9)	1498 (12.1)	1.87	1.74-2.01	<0.001	1.68	1.56-1.81	<0.001	1.43	1.31-1.56	<0.001
Dyslipidemia	2237 (17.7)	1772 (14.3)	1.53	1.43-1.65	<0.001	1.42	1.32-1.53	<0.001	1.24	1.14-1.35	<0.001
Dental caries	2060 (16.3)	1147 (9.3)	1.90	1.76-2.06	<0.001	1.67	1.54-1.81	<0.001	1.41	1.28-1.55	<0.001
Diabetes mellitus	1134 (9.0)	882 (7.1)	1.51	1.37-1.66	<0.001	1.28	1.16-1.41	<0.001	1.07	0.95-1.20	0.30
Mental diseases	1606 (12.7)	375 (3.0)	4.05	3.60-4.55	<0.001	3.25	2.88-3.67	<0.001	1.27	1.09-1.48	0.002
Asthma	763 (6.0)	407 (3.3)	1.84	1.63-2.09	<0.001	1.52	1.34-1.73	<0.001	1.02	0.87-1.20	0.79
Coronary artery diseases	525 (4.2)	267 (2.2)	2.12	1.82-2.47	<0.001	1.70	1.45-2.00	<0.001	1.18	0.97-1.43	0.09
Malignancies	543 (4.3)	313 (2.5)	1.89	1.64-2.19	<0.001	1.49	1.29-1.73	<0.001	1.09	0.91-1.30	0.36
Stroke	413 (3.3)	149 (1.2)	2.76	2.28-3.35	<0.001	2.08	1.71-2.54	<0.001	1.03	0.80-1.31	0.84
COPD	392 (3.1)	133 (1.1)	2.91	2.38-3.56	<0.001	2.12	1.72-2.61	<0.001	1.17	0.90-1.51	0.24

In this sensitivity analysis, we used unweighted regression instead of weighted regression; otherwise, see the caption of Table 3.

eTable 5. Association between social isolation and healthcare seeking behaviors (unweighted version)

	Participants with loneliness, n (%)	Participants without loneliness, n (%)	Model 1			Model 2			Model 3		
			AOR	95% CI	p value	AOR	95% CI	p value	AOR	95% CI	p value
Having a family doctor	5759 (45.6)	6448 (52.0)	0.90	0.85-0.94	<0.001	0.89	0.84-0.94	<0.001	0.89	0.83-0.95	0.001
Receiving annual health checkups	9084 (72.0)	9247 (74.6)	1.02	0.96-1.09	0.46	1.12	1.05-1.19	<0.001	1.33	1.23-1.44	<0.001
Receiving annual dental checkups	7293 (57.8)	7722 (62.3)	0.92	0.87-0.97	0.002	0.98	0.93-1.04	0.55	0.97	0.91-1.04	0.39
Vaccination in the previous year	5952 (47.1)	6112 (49.3)	1.09	1.04-1.15	0.001	1.13	1.07-1.19	<0.001	1.18	1.10-1.26	<0.001

In this sensitivity analysis, we used unweighted regression instead of weighted regression; otherwise, see the caption of Table 4.
